# Twenty-Four-Hour Contact Lens Sensor Monitoring of Aqueous Humor Dynamics in Surgically or Medically Treated Glaucoma Patients

**DOI:** 10.1155/2019/9890831

**Published:** 2019-01-27

**Authors:** Chiara Posarelli, Pierluigi Ortenzio, Antonio Ferreras, Mario Damiano Toro, Andrea Passani, Pasquale Loiudice, Francesco Oddone, Giamberto Casini, Michele Figus

**Affiliations:** ^1^Ophthalmology, Department of Surgical, Medical, Molecular Pathology and of Critical Area, University of Pisa, Pisa, Italy; ^2^Department of Ophthalmology, Miguel Servet University Hospital, Zaragoza, Spain; ^3^Eye Clinic, University of Catania, Catania, Italy; ^4^Ophthalmology, IRCCS G.B.Bietti Foundation, Rome, Italy

## Abstract

**Aim:**

This study assessed the 24 h circadian rhythm of intraocular pressure (IOP) using a contact lens sensor in three groups of patients with open-angle glaucoma.

**Methods:**

This study was a monocentric, cross-sectional, nonrandomized, prospective, pilot study. Eighty-nine patients were enrolled: 29 patients previously underwent an Ex-PRESS mini glaucoma device procedure (Group 1), 28 patients previously underwent Hydrus microstent implantation (Group 2), and 32 patients were currently being treated medically for primary open-angle glaucoma (Group 3). Circadian rhythm patterns were considered with five circadian indicators: fluctuation ranges, maximum, minimum, acrophase (time of peak value), and bathyphase (time of trough value). A two-tailed Mann–Whitney *U*-test was used to evaluate differences between groups.

**Results:**

All subjects exhibited a circadian rhythm and a nocturnal pattern. The signal fluctuation range was significantly smaller in the surgical groups than in the medically treated group (Group 1 vs. Group 3, *p*=0.003; Group 2 vs. Group 3, *p*=0.010). Subjects who underwent the Ex-PRESS procedure (Group 1) exhibited significant differences compared with the drug therapy group (Group 3) with regard to the minimum value (*p*=0.015), acrophase (*p*=0.009), and bathyphase (*p*=0.002). The other circadian indicators were not significantly different among groups.

**Conclusions:**

Patients who underwent IOP-lowering surgery had an intrinsic nyctohemeral rhythm. Both surgical procedures, Ex-PRESS and Hydrus, were associated with smaller signal fluctuations compared with medical treatment.

## 1. Introduction

Glaucoma is the principal cause of irreversible vision loss and the second leading cause of blindness worldwide. The number of people aged 40–80 years with glaucoma was estimated to be 64.3 million in 2013, and this number is expected to increase to 76.0 million by 2020 and 111.8 million by 2040 [1]. The management of glaucoma focuses on lowering intraocular pressure (IOP), which still remains the rational proven treatment method [2]. Current management of glaucoma includes medical, laser, or surgical reduction of IOP to a predetermined target. The follow-up of glaucoma patients with single IOP measurements using Goldmann applanation tonometry (GAT), although quick and economically convenient, often does not adequately reflect the untreated IOP characteristics or the quality of treated IOP control over a 24 h period [3]. IOP is not a static parameter but undergoes dynamic changes. Several options for monitoring 24 h IOP are available. The most popular method to assess an IOP curve is based on a series of single IOP measurements during a determined period, which requires consecutive visits or hospitalization. Contact lens sensors (CLSs) could be a better choice, particularly for research purposes. A CLS provides 288 measurements over 24 h by sensing geometrical changes at the corneoscleral junction every 5 minutes. The main limitation of this approach is that data are given in arbitrary units (mVEq) instead of standard tonometry pressure values (mmHg). This device can record signal fluctuations in an outpatient setting for up to 24 h, including during undisturbed sleep. It provides a paradigmatic change in IOP evaluation, both clinically and experimentally. Moreover, applying modeling to CLS output could markedly simplify data interpretation by providing a few circadian indicators [4].

IOP reduction remains the mainstay of glaucoma management. The conventional stepwise algorithm involves medical treatment, laser trabeculoplasty, and surgical techniques. It is generally established that surgery offers better IOP control than medical therapy, but surgery may be associated with complications and failures. For this reason, surgery is usually reserved for advanced or clearly progressive glaucoma. In recent years, great efforts have been made to enhance the safety and efficacy of conventional surgery. This is currently a rapidly evolving field, with the development of an increasing number of innovative procedures often working on completely different bases. In the present study, we used a CLS to characterize the 24 h circadian signal rhythm in 29 patients that had undergone Ex-PRESS mini glaucoma device implantation, in 28 patients that had undergone Hydrus microstent implantation, and in 32 primary open-angle glaucoma (POAG) patients that were being treated medically. We evaluated the patterns of circadian signal fluctuation curves and five circadian indicators: fluctuation range, maximum, minimum, acrophase (time of peak value), and bathyphase (time of trough value).

## 2. Materials and Methods

### 2.1. Population

This was a monocentric, cross-sectional, nonrandomized, prospective, pilot study performed in accordance with the tenets of the Declaration of Helsinki. The Institutional Review Board of the Azienda Ospedaliero-Universitaria Pisana approved the project, and all ruled-in subjects signed the informed consent after appropriate explanation of the procedure and possible consequences. Patients were consecutively recruited from February 2016 through November 2017 in the Ophthalmology Unit, Department of Surgery, at the Azienda Ospedaliero-Universitaria of Pisa. Eighty-nine patients were enrolled and divided into three groups based on their treatment history: Group 1 previously underwent Ex-PRESS mini glaucoma device implantation (29 patients, CLS applied on the operated eye), Group 2 previously underwent Hydrus implantation (28 patients, CLS applied on the operated eye), and Group 3 was currently undergoing medical treatment with two drugs (beta blockers and prostaglandin analogues on 32 patients, CLS applied on the right eye). Surgery for Group 1 and Group 2 had been performed at least 2 years before by the same expert surgeon and did not require any additional IOP-lowering drug at the time of selection. Participants that did not sign the informed consent, did not agree to wear the CLS, or had more than one surgical procedure previously were excluded from the analysis. All patients were between 18 and 80 years old and had a mean deviation on standard automated perimetry (SAP) lower than −4 dB.

The Ex-PRESS device (Alcon Laboratories, Fort Worth, TX, USA) is an ab externo anterior filtering procedure that enhances aqueous humor outflow. It is a stainless steel, nonvalved device placed under a partial-thickness scleral flap that diverts aqueous humor from the anterior chamber to the subconjunctival space to create a filtration bleb. The efficacy and safety of the device are comparable to those of the current gold standard trabeculectomy, while reducing both intraoperative and postoperative complications [5]. The magnitude of IOP reduction with the Ex-PRESS mini glaucoma device is highly valuable in eyes with advanced glaucoma where the IOP target is set as low as possible to slow down further progression of the disease [6–9].

The Hydrus microstent (Ivantis, Irvine, CA, USA) is an ab interno procedure that restores the natural pathway. It is a Schlemm canal (SC) scaffold, a 8 mm long nitinol device, that is inserted with a delivery cannula into the SC. It is often associated with cataract surgery. The idea of this device is to dilate the SC and provide a scaffold that allows the aqueous humor to access multiple collector channels. The device increases the aqueous outflow through the trabecular meshwork. It is longer than other devices so it maintains a longer length of the SC open with greater probability to involve more collector channels. It is implanted through a clear corneal incision under gonioscopic view [6, 10]. Preliminary results support a favorable effectiveness and safety profile after 12 to 24 months [11, 12]. Inserting the Hydrus microstent is considered a minimally invasive glaucoma surgery (MIGS) technique, and its intrinsic safety and efficacy may encourage an earlier transition to surgery and reduce the latency of topical glaucoma medication management with its associated compliance and intolerance issues.

### 2.2. Measurement of 24 h IOP with CLS

All participants underwent a screening visit including a comprehensive ophthalmologic examination, review of medical history, keratometry, ultrasound pachymetry, slit-lamp biomicroscopy, and gonioscopy. Baseline IOP was measured with GAT, both before starting and after ending the 24 h IOP monitoring session; each session was initiated at 9 o'clock and completed after 24 h.

Signal monitoring was conducted with a CLS (Triggerfish; Sensimed AG, Lausanne, Switzerland). Lens size was selected among the three available base curves (8.4 mm steep, 8.7 mm medium, and 9 mm large). The device comprises a contact lens capable of recording qualitative circadian pressure-related profiles over a 24 h period under physiologic conditions. A strain gauge embedded in a soft silicon contact lens (sensor) captured spontaneous circumferential changes of the corneoscleral area. The data were transmitted to a portable recorder via wireless telemetry. Three hundred data points were acquired every 30 seconds, repeated every 5 minutes, providing a total of 288 data points at the end of 24 h. Along with the hardware (CLS, antenna, and black box), software is provided to graphically visualize the raw data. The device does not measure pressure but rather measures geometrical changes: for this reason, the data are displayed as an mVEq unit that corresponds to mV. Many studies have validated the good correlation of this measurement system with manometric pressure values [4, 13, 14]. The range of fluctuations (or amplitude, mVEq) was defined as the difference between the maximum value (or acrophase, mVEq) and the minimum value (or bathyphase, mVEq) over 24 h; signal fluctuation was calculated for each patient, and then ranges in fluctuations were compared between groups.

The patients had no restrictions during the 24 h recording time as the design and purpose of this research was to detect differences in conditions that could be representative of everyday life as much as possible. Patients pursued their routine activities without controlling their sleep. Nonetheless, participants were instructed to take notes in a diary of meaningful facts that could affect measurement and IOP rhythm itself, such as the time they went to bed, the time they woke up, and the time medication was applied or consumed.

### 2.3. Modeling Circadian Pattern

Starting from raw data obtained over 24 h, mathematical estimation of the signal was made using a robust nonlinear least-squares model based on a generalized Fourier transform (1st level):

(1) $$\begin{array}{c} y=a_{0}+a_{1}\cos\left(\omega x\right)+b_{1}\sin\;\;\omega x, \end{array}$$

where *y* is the observed signal (mVEq); *a*_0_, *a*_1_, and *b*_1_ are regression coefficients; *ω* is the angular frequency or pulsatance, known as the rate of change of the phase of a sinusoidal waveform (e.g., in oscillations and waves) or as the rate of change of the argument of the sine function; and *x* is an independent variable, which is the serial number of recording.

We applied robust fitting to minimize the influence of outliers using the least absolute residual (LAR) method: this analysis minimizes the absolute difference of residuals rather than the square difference, and therefore, extreme values have a smaller influence on the fit. Goodness of fit was evaluated with single parameter indicators: *r*^2^ (coefficient of determination), *r*^2^_adj_ (adjusted *r*^2^), and root-mean-square error (RMSE).

A general Fourier transform was applied to data distributions of all 24 patients to classify them into pattern groups based on the recent literature: [15–20] diurnal, nocturnal, and unclassifiable. The diurnal pattern was defined when the maximum peak occurred during the diurnal/wakefulness period and the mean diurnal amplitude was higher than the nocturnal amplitude. The nocturnal pattern was defined when the maximum peak occurred during the nocturnal/sleep period and the mean nocturnal amplitude was higher than diurnal amplitude. The unclassifiable pattern was defined when acrophase did not occur in the same period as the maximum mean amplitude.

### 2.4. Statistical Analysis

This was a pilot explorative study. Standard descriptive statistics were used both within and between groups. Data are expressed as mean ± standard deviation (SD) where appropriate. Categorical variables are described in terms of percentage. Outlier values are defined according to Tukey's range test as elements with more than 1.5 interquartile ranges above the upper quartile (75%) or below the lower quartile (25%). The data-filling method for outliers was performed by replacing the outlier data with the lower threshold value for elements smaller than the lower threshold (lower quartile) and with the upper threshold value for elements larger than the upper threshold (upper quartile). Statistical significance was defined at *p* < 0.05. Bonferroni's correction for multiple comparisons was applied when appropriate (*p* < 0.017). The Kruskal–Wallis test was the first step to assess statistically significant differences among the three groups, and then a two-tailed Mann–Whitney *U*-test for independent samples was performed considering two groups at a time. Analysis was performed to compare demographic and clinical data (age (years), GAT-IOP before and after application of CLS (mmHg), mean deviation of SAP (dB), pattern standard deviation of SAP (dB), and central corneal thickness (*µ*m)), and nyctohemeral curve parameters (*ω* coefficient, range of fluctuation (mVEq), maximum (mVEq), minimum (mVEq), acrophase (h), and bathyphase (h)). GAT-IOP before and after application of CLS (mmHg) was investigated within groups using the two-tailed Wilcoxon signed-rank test. All analyses were performed using the academic analytic software package MATLAB (MATLAB R2017a 9.2.0.538062; The MathWorks Inc., Natick, MA, USA).

## 3. Results

### 3.1. Ophthalmic Data

The demographics and clinical data of the three groups are shown in Table 1. The mean age of our population was 73.96 years, with no difference between groups. The patients comprised 38 men and 51 women. Baseline IOP before CLS application and that after application are shown in Table 1. The decrease in IOP after CLS removal was significant only in Group 1 (*p*=0.031). NSAP-mean deviation and central corneal thickness were not significantly different at baseline among the groups. The pattern standard deviation of SAP was greater in Group 1 than in Group 2 (*p*=0.01).

### 3.2. Circadian IOP Patterns

**Figure 1:**
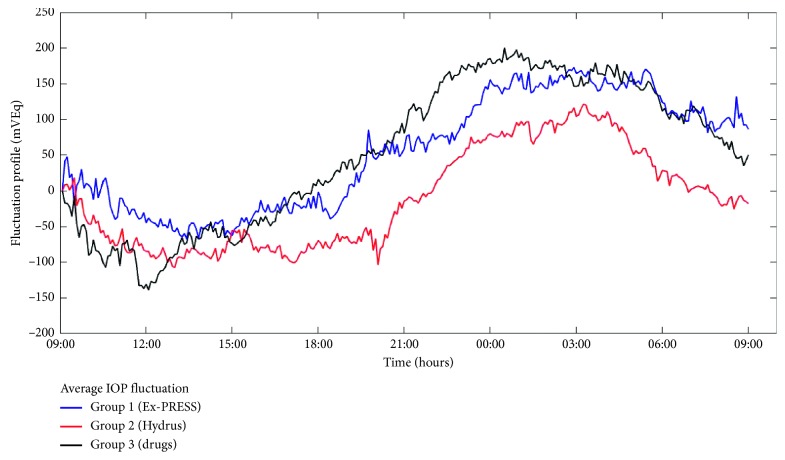
Circadian signal fluctuation range in each group. A nocturnal pattern is observed for all three curves. Group 3 shows a larger signal fluctuation range than the surgical groups (Groups 1 and 2). Group 3 peaks earlier than Group 1 in both acrophase and bathyphase. Minimum (mVEq) is significantly deeper in Group 3 compared with Group 1. These indicators taken together implicate a smoother curve for Group 1. Fluctuation profile's unit (mVEq) is arbitrarily assigned by the contact lens sensor.

**Figure 2:**
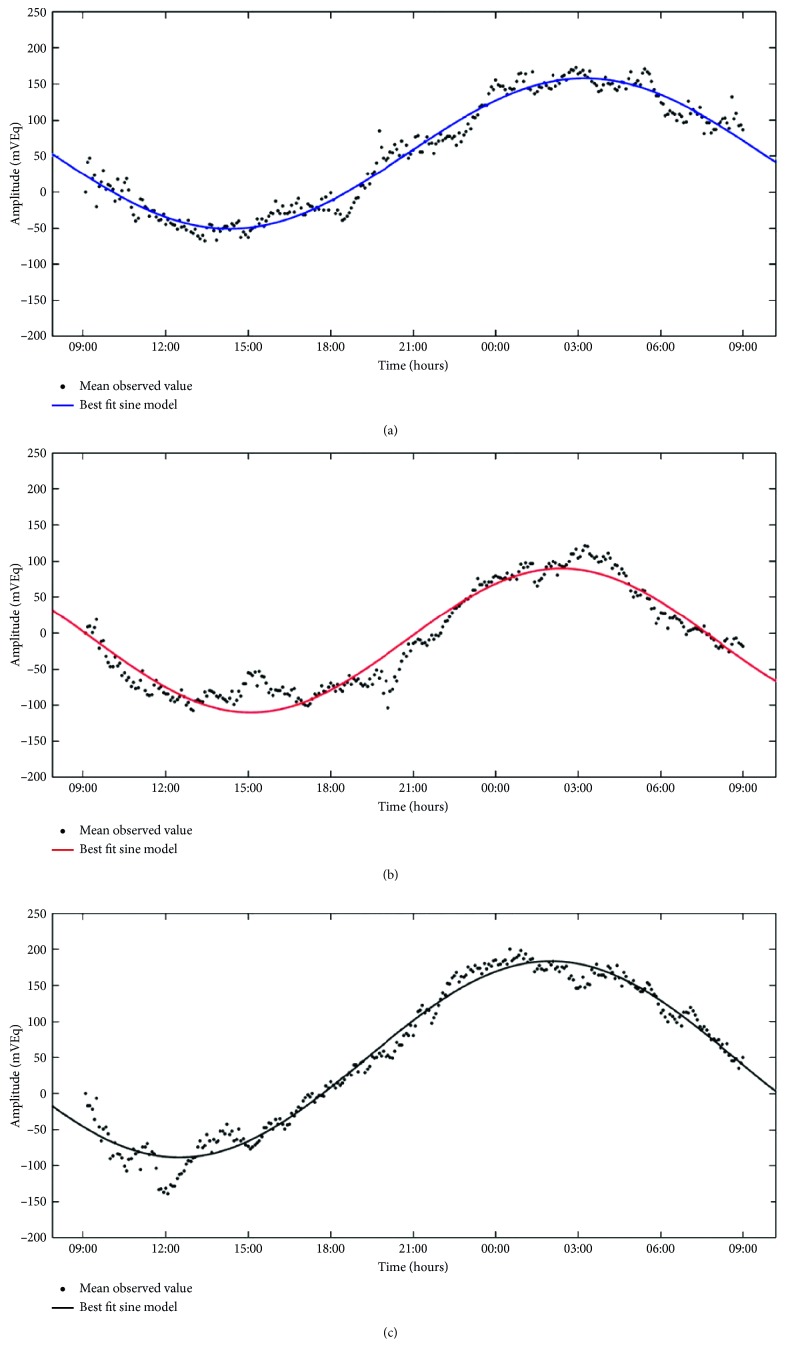
Examples of mean sine modeling of each group according to a robust nonlinear least-squares model based on a generalized Fourier transform (Section 3). Dots indicate the mean of observed values, and the solid line indicates the modeling function. Goodness of fit was evaluated by the sum of squares due to error (SSE), coefficient of determination (*r*^2^), adjusted coefficient of determination (adjusted *r*^2^), and root-mean-square error (RMSE). (a) Group 1 (Ex-PRESS) coefficients: *a*_0_ = −10.15; *a*_1_ = 12.45; *b*_1_ = −99.1; *ω* = 0.021; goodness of fit: SSE: 1.089*e* + 05; *r*^2^: 0.921; adjusted *r*^2^: 0.920; RMSE: 19.58. (b) Group 2 (Hydrus) coefficients: *a*_0_ = −10.15; *a*_1_ = 12.45; *b*_1_ = −99.1; *ω* = 0.025; goodness of fit: SSE: 1.089*e* + 05; *r*^2^: 0.921; adjusted *r*^2^: 0.920; RMSE: 19.58. (c) Group 3 (drugs) coefficients: *a*_0_ = 47.43; *a*_1_ = −93.41; *b*_1_ = −99.03; *ω* = 0.021; goodness of fit: SSE: 7.21*e* + 04; *r*^2^: 0.976; adjusted *r*^2^: 0.975; RMSE: 15.93.

Circadian rhythms were successfully recorded for a 24 h period in all patients (Figure 1). Using equation (1), we demonstrated a circadian rhythm by evaluating coefficient *ω*. If 288 measurements were obtained in 24 h (i.e., *τ* = 288) and pulsatance was defined as *ω* = 2*π*/*τ*, then *ω*_expected_ = 0.0218 (Figure 2).

Table 2 shows that recorded pulsatance in Group 1 (Ex-PRESS) was 0.021 ± 0.003 (93.99% of *ω*_expected_; *r*^2^ = 0.651 – 0.996). In Group 2 (Hydrus), pulsatance was 0.025 ± 0.005 (exceeding 12.92% of *ω*_expected_; *r*^2^ = 0.754 – 0.996). In Group 3 (medication), pulsatance was 0.021 ± 0.004 (94.29% of *ω*_expected_; *r*^2^ = 0.815 – 0.995). Moreover, no statistically significant differences were detected between groups (Table 2).

According to pattern classification, 100% of patients belonged to the nocturnal group.

### 3.3. Nyctohemeral Indicators

The fluctuation range in both surgical groups was significantly different from that in the medically treated group (Figure 1). No significant differences in the five nyctohemeral indicators were detected between the surgical groups. Differences between maximum values were not significant. Patients who underwent Ex-PRESS mini glaucoma device implantation (Group 1) had a significantly different minimum value (*p*=0.015), acrophase (*p*=0.009), and bathyphase (*p*=0.002) compared with patients in drug therapy (Group 3).

**Figure 3:**
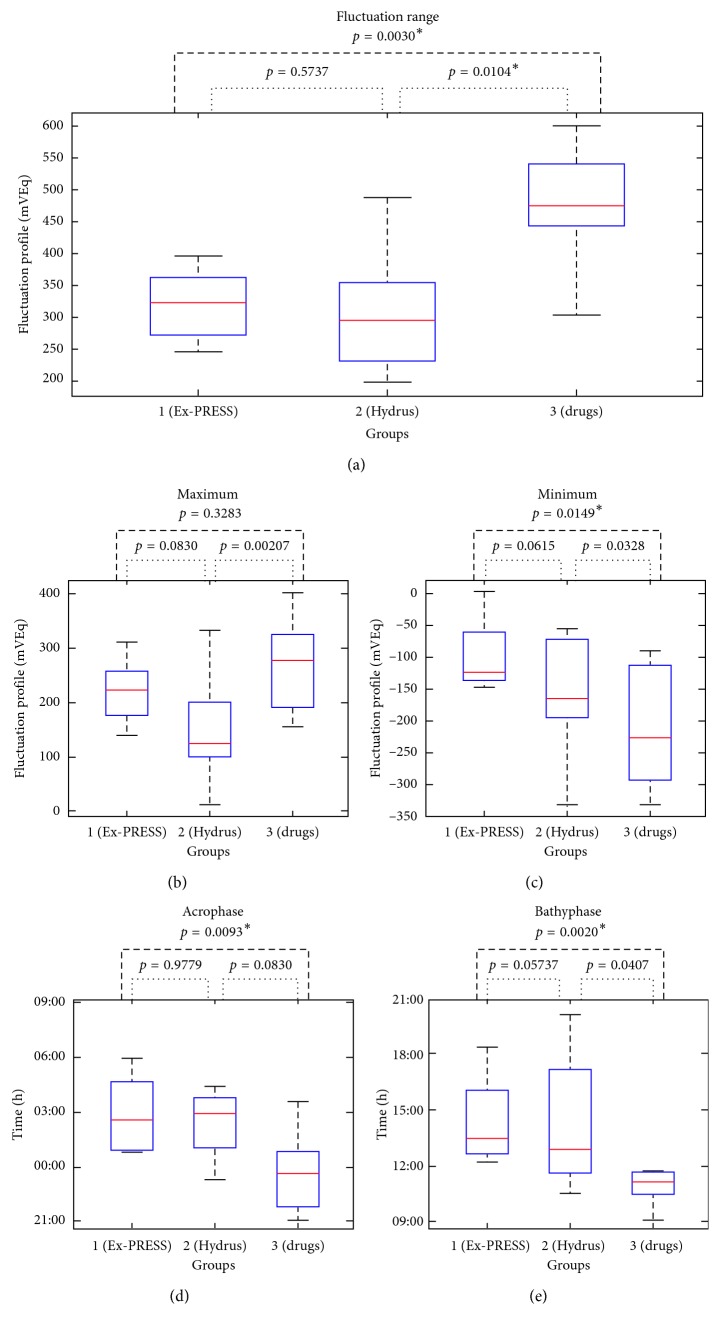
Boxplot and statistics of circadian indicators. (a) Fluctuation range. (b) Maximum. (c) Minimum. (d) Acrophase (time to peak). (e) Bathyphase (time to peak). mVEq, millivolt equivalent, an arbitrary unit provided by the contact lens sensor; h, hours; ^*∗*^statistically significant values by the Mann–Whitney *U*-test with Bonferroni's correction for multiple comparisons (*p* < 0.017).

All data concerning circadian indicators are shown in Table 2 and Figure 3.

## 4. Discussion

We used a CLS to characterize the 24 h circadian rhythm of IOP in patients that underwent the Ex-PRESS procedure and Hydrus microstent implantation and POAG patients that were treated medically. Currently, the most common method for studying IOP rhythm in glaucoma patients is through a diurnal tension curve. Nighttime IOP monitoring requires hospitalization or sleep laboratories, and not only it is costly but it does not provide reliable data due to patients' awakening during the sleep period, which potentially introduces stress-related artifacts. Due to the lack of available IOP-recording techniques that do not interfere with the sleep cycle, IOP rhythms have been quite unexplored until recently [21–23].

IOP is a dynamic parameter with an individual rhythm, and potentially informative indicators, such as IOP fluctuation and peak IOP, are still neglected in clinical practice. Moreover, the effects of IOP-lowering interventions on such measures are largely uninvestigated. Maximum IOP peak is a case in point: it is widely accepted that the highest IOP values occur at night, outside office hours [24, 25]. A telemetric sensor may overcome this limitation by providing continuous 24 h IOP monitoring. We used a Sensimed Triggerfish CLS to record 288 measurements over 24 h. The main limitation of such a device is that the output is not IOP (mmHg) itself but an equivalent of electric voltage (mVEq) induced by conformational ocular dimensional changes at the corneal-scleral junction that are transmitted to the recorder [14, 21, 22].

We considered the patterns of circadian signal curves and five circadian indicators: fluctuation ranges, maximum, minimum, acrophase, and bathyphase. Actually, the definition of target IOP is dampening of its fluctuations throughout the day, as these daily variations are identified as a significant and independent risk factor for glaucomatous progression [3, 23, 26]. Recently, Tojo et al. [18] using the same CLS device (Sensimed Triggerfish) reported that short-term IOP fluctuations are associated with long-term IOP fluctuations. They found that the long-term parameter IOP difference (difference between maximum and minimum IOP) and IOP peak (maximum IOP) significantly correlated with short-term IOP fluctuations (Spearman's *r* = 0.326, *p*=0.021, and Spearman's *r* = 0.433, *p*=0.002, respectively). These results strengthen both the need for a deeper investigation of circadian parameters in a continuous 24 h IOP monitoring and an update of the guidelines for surgical and medical management of glaucoma.

The main findings of the present study were that the signal fluctuation range was significantly smaller in the surgery groups compared to the medically treated group. We also observed that the minimum IOP was higher in the Ex-PRESS group than in the medically treated group, but maximum IOP did not differ between them.

Compared with the medication group, the Hydrus microstent was more effective only in reducing the signal fluctuation range. All of the other circadian indicators were not significantly different between the Hydrus microstent and the other groups. These results are not surprising because the Hydrus microstent is an MIGS device, whose target IOP is in the mid-teens, but there is a growing body of evidence suggesting that MIGS might be a viable initial treatment option [10, 11].

Furthermore, our results indicated that all patients had a nyctohemeral rhythm with a nocturnal acrophase, consistent with the other CLS studies [15–17, 20], and provided us with the possibility to compare groups without any pattern biases, particularly concerning more easily affected data: circadian indicators. A circadian rhythm with a nocturnal acrophase was identified in medically treated POAG in several previous reports [16, 27–29]. Mansouri et al. [28] reported a repeatable nocturnal acrophase in 62.9% of patients, and Mansouri and Shaarawy [30] reported it in 69% of patients. Such a difference could be explained by the larger study population and extreme variety of IOP-lowering drugs used, both in fixed combination and as monotherapy. Our results were much closer to those reported by Agnifili et al. [16], who found a nocturnal pattern in 90% of patients medically controlled with prostaglandin analogues. Prostaglandin analogues uniformly decrease IOP throughout the circadian cycle [31, 32]. Thus, in patients controlled with such pharmacologic therapy, peaks may occur more commonly during the night, especially the first part of the night. Recent findings [33] indicated that prostaglandin analogues do not affect acrophase and amplitude compared with other classes of ocular hypotensive medications. Accordingly, we could consider our pharmacologically treated group as a homogeneous sample despite the use of different IOP-lowering medications.

In addition, we found that the surgical groups exhibited a nocturnal pattern. Few studies have assessed a nocturnal nyctohemeral rhythm of IOP using a CLS [15, 17, 34]. Tojo et al. [15] examined the effects of SLT on IOP fluctuations in normal-tension glaucoma patients. They found that SLT treatment significantly decreased IOP and decreased IOP fluctuations during the nocturnal periods. Aptel et al. [17] studied a population of POAG patients after SLT and medication washout. They reported that SLT did not significantly change the characteristics of the 24 h pattern and that 100% of patients had a nocturnal acrophase after the medication washout.

Regarding temporal parameters, acrophase and bathyphase, a difference was observed between Group 1 and Group 3. Group 1's acrophase peaked later in the night compared with Group 3, as did bathyphase. An invasive surgery procedure (Ex-PRESS) could reduce the slope of the IOP curve. Beyond reducing the IOP fluctuation range itself, the Ex-PRESS could change the mechanism by which IOP fluctuated within a day by lowering the IOP slope and without interfering with the circadian rhythm. The use of the robust nonlinear least-squares model based on a generalized Fourier transform allowed us to study all patients with a highly significant coefficient of determination, without any of the a priori assumptions used in other modeling techniques [16, 17, 19, 28]. Although this could be considered an unremarkable result, it is not useless because it reveals that therapy, both medical and surgical, did not elicit an intrinsic natural circadian rhythm. Nevertheless, further studies are needed to clarify this point.

A potential limitation is the corneal effect of wearing a CLS and the efficacy of the 24 h measurements. We also cannot exclude the possibility that artifacts like corneal swelling may have affected our results. Although corneal swelling is a commonly observed phenomenon when using a CLS, Freiberg et al. [35] showed that continuous IOP monitoring does not seem to be affected by differences in the corneal thickness occurring overnight when wearing a CLS. Another weakness of our study is the absence of a conversion table for mVEq into mmHg units. The CLS recorded the relative signal fluctuations from the starting point. The precise relationship between the CLS output and absolute data given by standard tonometry remains unknown. Actually, the inability to convert to mmHg does not affect our data. On the one hand, we could not directly compare our results with those of previous studies, specifically older studies, while on the other hand, new parameters that can be relatively evaluated even with conventionally agreed on arbitrary units are gaining more and more popularity given their importance, both experimentally and clinically, compared with absolute IOP measures themselves.

In conclusion, this study is the first to compare subjects that underwent an Ex-PRESS procedure, patients implanted with a Hydrus microstent, and medically treated POAG patients using a CLS for 24 h continuous IOP monitoring. Based on our results, both the Ex-PRESS and Hydrus microstent appear to be better able to manage aqueous humor dynamics and maybe also IOP fluctuations over a 24 h period than medical treatment.

## Figures and Tables

**Table 1:** Demographics and clinical data of subjects.

	Ex-PRESS (Group 1)	Hydrus (Group 2)	Drug therapy (Group 3)	Group 1 vs. Group 2 (*p* value)	Group 1 vs. Group 3 (*p* value)	Group 2 vs. Group 3 (*p* value)
Age (years)	71.12 ± 11.42	77.75 ± 4.62	73.25 ± 4.78	0.14	0.88	0.07
Sex (M/F)	17/12 (29)	11/17 (28)	11/21 (32)	—	—	—
Eye laterality (right/left)	17/12 (29)	9/19 (28)	13/19 (32)	—	—	—
GAT-IOP before CLS application (mmHg)	17.62 ± 3.42	16.62 ± 3.11	17.25 ± 3.11	0.59	0.89	0.59
GAT-IOP after CLS removal (mmHg)	14.25 ± 2.55	15.65 ± 1.60	15.87 ± 3.90	0.23	0.45	0.78
CCT (*µ*m)	474 ± 47.27	515.12 ± 20.25	528.12 ± 18.43	0.082	0.016	0.15
MD of SAP (dB)	−14.37 ± 9.74	−5.00 ± 2.22	−6.07 ± 2.19	0.021	0.038	0.38
PSD of SAP (dB)	9.42 ± 4.61	4.21 ± 2.13	6.38 ± 3.05	**0.015** ^*∗*^	0.16	0.13

Data are expressed as mean ± SD, except for sex and eye laterality. SD, standard deviation; *p*, probability value of statistical hypothesis testing; GAT-IOP, Goldmann applanation tonometry intraocular pressure; CLS, contact lens sensor; CCT, central corneal thickness; MD, mean deviation; SAP, standard automated perimetry; PSD, pattern standard deviation; mmHg, millimeters of mercury; dB, decibel. ^*∗*^Significant values by the Mann–Whitney *U*-test with Bonferroni's correction for multiple comparisons (*p* < 0.017).

**Table 2:** Circadian indicators.

	Ex-PRESS (Group 1)	Hydrus (Group 2)	Drug therapy (Group 3)	Group 1 vs. Group 2 (*p* value)	Group 1 vs. Group 3 (*p* value)	Group 2 vs. Group 3 (*p* value)
Observed circadian pattern, *n*/*n* (%)	29/29 (100)	28/28 (100)	32/32 (100)	—	—	—
Nocturnal pattern recorded, *n*/*n* (%)	29/29 (100)	28/28 (100)	32/32 (100)	—	—	—
Coefficient *ω*	0.021 ± 0.00	0.025 ± 0.01	0.021 ± 0.00	0.26	0.62	0.13
Fluctuation range (mVEq)	319.32 ± 53.26	305.75 ± 9.44	477.70 ± 92.74	0.57	**0.003** ^*∗*^	**0.010** ^*∗*^
Maximum (mVEq)	221.22 ± 60.04	149.39 ± 97.49	267.97 ± 85.38	0.08	0.33	0.021
Minimum (mVEq)	−98.11 ± 58.86	−156.36 ± 91.63	−209.72 ± 97.53	0.06	**0.015** ^*∗*^	0.033
Acrophase (h)	04 : 53 ± 1 : 59	04 : 01 ± 2 : 51	22 : 26 ± 4 : 58	0.98	**0.009** ^*∗*^	0.083
Bathyphase (h)	14 : 19 ± 2 : 22	14 : 11 ± 3 : 23	11 : 12 ± 1 : 18	0.57	**0.002** ^*∗*^	0.041

Data are expressed as mean ± SD, except for observed circadian pattern and eye nocturnal pattern recorded. SD, standard deviation; *p*, probability value of statistical hypothesis testing; *n*, number of patients; mVEq, millivolt equivalent, an arbitrary unit provided by the contact lens sensor; h, hours. ^*∗*^Significant values by the Mann–Whitney *U*-test with Bonferroni's correction for multiple comparisons (*p* < 0.017).

## Data Availability

The charts of every patient and all the measurements of the contact lens sensor used to support the findings of this study are restricted by the Institutional Review Board of the Azienda Ospedaliero-Universitaria Pisana in order to protect patient privacy. Data are available from Michele Figus, MD, PhD, figusmichele@gmail.com, for researchers who meet criteria for access to confidential data.
